# Detection of respiratory viruses directly from clinical samples using next‐generation sequencing: A literature review of recent advances and potential for routine clinical use

**DOI:** 10.1002/rmv.2375

**Published:** 2022-07-01

**Authors:** Xinye Wang, Sacha Stelzer‐Braid, Matthew Scotch, William D. Rawlinson

**Affiliations:** ^1^ Virology Research Laboratory Serology and Virology Division (SAViD) NSW Health Pathology Prince of Wales Hospital University of New South Wales Sydney New South Wales Australia; ^2^ School of Medical Sciences Faculty of Medicine University of New South Wales Sydney New South Wales Australia; ^3^ Kirby Institute University of New South Wales Sydney New South Wales Australia; ^4^ Biodesign Center for Environmental Health Engineering Biodesign Institute Arizona State University Tempe Arizona USA

**Keywords:** barriers, clinical use, next‐generation sequencing (NGS), respiratory viruses

## Abstract

Acute respiratory infection is the third most frequent cause of mortality worldwide, causing over 4.25 million deaths annually. Although most diagnosed acute respiratory infections are thought to be of viral origin, the aetiology often remains unclear. The advent of next‐generation sequencing (NGS) has revolutionised the field of virus discovery and identification, particularly in the detection of unknown respiratory viruses. We systematically reviewed the application of NGS technologies for detecting respiratory viruses from clinical samples and outline potential barriers to the routine clinical introduction of NGS. The five databases searched for studies published in English from 01 January 2010 to 01 February 2021, which led to the inclusion of 52 studies. A total of 14 different models of NGS platforms were summarised from included studies. Among these models, second‐generation sequencing platforms (e.g., Illumina sequencers) were used in the majority of studies (41/52, 79%). Moreover, NGS platforms have proven successful in detecting a variety of respiratory viruses, including influenza A/B viruses (9/52, 17%), SARS‐CoV‐2 (21/52, 40%), parainfluenza virus (3/52, 6%), respiratory syncytial virus (1/52, 2%), human metapneumovirus (2/52, 4%), or a viral panel including other respiratory viruses (16/52, 31%). The review of NGS technologies used in previous studies indicates the advantages of NGS technologies in novel virus detection, virus typing, mutation identification, and infection cluster assessment. Although there remain some technical and ethical challenges associated with NGS use in clinical laboratories, NGS is a promising future tool to improve understanding of respiratory viruses and provide a more accurate diagnosis with simultaneous virus characterisation.

**Abbreviations**
AdV: adenovirus
ARI: acute respiratory infection
Ct: cycle threshold
DFA: direct fluorescent antibody
FDA: Food and Drug Administration
HBoV: Human bocavirus
HMPV: human metapneumovirus
HRV: human rhinovirus
IFV: Influenza virus
LRTIs: lower respiratory tract infections
MERS‐CoV: Middle East respiratory syndrome coronavirus
mNGS: metagenomic next‐generation sequencing
NAAT: nucleic acid amplification test
NGS: next‐generation sequencing
PIV: parainfluenza virus
RADT: rapid antigen detection test
RSV: respiratory syncytial virus
SARS‐CoV: severe acute respiratory syndrome coronavirus
SARS‐CoV‐2: severe acute respiratory syndrome coronavirus 2
URTIs: upper respiratory tract infections
WGS: whole genome sequencing

## 1 INTRODUCTION

Acute respiratory infections (ARIs) are the most common infectious disease associated with high mortality and morbidity rates in humans throughout the world.^1^ In developing countries, the disease burden resulting from ARIs is 10–50 times higher than in developed countries.^2^ It is estimated that ARI is responsible for more than four million deaths globally each year.^3^ Approximately 80% of ARIs with a diagnosed aetiology are caused by viral pathogens,^4^ such as influenza viruses (IFVs), respiratory syncytial viruses (RSVs), parainfluenza viruses (PIVs), adenoviruses (AdVs), and human rhinoviruses (HRVs).^5^, ^6^ Although most diagnosed ARIs are viral in origin, the underlying pathogens often remain unknown, since the respiratory viral pathogens other than influenza viruses have received little attention to date.^7^ Every year, influenza viruses alone are estimated to cause infections in approximately 9% of the world's population, three to five million severe cases, and 290,0000–650,000 influenza‐related deaths.^8^ One of the leading causes of acute lower respiratory tract infections (LRTIs) in infants and children is RSV, which causes millions of hospitalisations and over 66,000 deaths per year globally.^9^, ^10^, ^11^ Other common respiratory viral pathogens, such as HRV and AdV cause relatively lower mortality rates worldwide when compared to IFV infections, but such infections can still lead to significant economic losses.^12^, ^13^ Furthermore, outbreaks or pandemics related to emerging respiratory viruses have occurred continuously over the past decades. These include severe acute respiratory syndrome coronavirus (SARS‐CoV), IFV (H5N1, H7N9, and H1N1), Middle East respiratory syndrome coronavirus (MERS‐CoV), and most recently severe acute respiratory syndrome‐coronavirus‐2 (SARS‐CoV‐2), all of which pose significant threats to public health.

ARIs are classified as upper respiratory tract infections (URTIs) (e.g., rhinitis, pharyngitis, and laryngitis) or LRTIs (e.g., bronchitis, bronchiolitis, and pneumonia). The predominant involvement of upper or lower airways is dependent upon many factors, including virus type, virus strain, and host. For example, a variety of respiratory viruses have a tendency for LRTI and cause pneumonia, including RSVs, PIVs (e.g., serotype 3), certain types of AdVs (e.g., type 3, 7, and 21), and certain types of IFVs (e.g., influenza A and C virus).^6^, ^9^, ^14^, ^15^, ^16^ Infection of the lower airways is more likely than URTIs to cause severe illness and death.^17^ The early, rapid, and accurate identification of respiratory viral pathogens using advanced laboratory methods is critical for selecting appropriate treatment plans, saving lives, developing new vaccines and drugs, as well as containing potential outbreaks.

Laboratory methods currently used in clinical laboratories for the diagnosis of respiratory virus infections include nucleic acid amplification tests (NAAT), direct fluorescent antibody testing (DFA), and rapid antigen detection tests (RADT).^18^ Among these laboratory methods, NAATs have been considered as the diagnostic reference standard in clinical laboratories due to their high sensitivity and specificity, and no requirement for calibration.^19^ However, one common pitfall associated with all these methods includes limits to the number of targets or targeting of conserved regions of the viral sequence or virus typing, which may result in atypical or novel viruses evading detection (Table S1 provides a comparison between the above laboratory methods). Hence, the introduction of next‐generation sequencing technologies in clinical settings may break these limitations and supplement conventional molecular methods to provide more valuable clinical information.^20^, ^21^

Next‐generation sequencing (NGS), also known as massively parallel, deep, or high‐throughput DNA sequencing, is a method that allows simultaneously sequence millions of small DNA fragments in parallel.^22^ These NGS technologies incorporate many advantages over conventional molecular methods, such as the provision of higher yield, faster turnaround time, and more comprehensive genomic information. With the reduction in the cost of genome sequencing over time, more laboratories have been able to access these technologies.^23^ In addition, three main methods based on NGS technologies are currently used to sequence viral genomes (whole‐genome), including PCR amplicon sequencing, target enrichment sequencing, and metagenomic sequencing.^24^, ^25^, ^26^, ^27^, ^28^ Each method has its own strengths that enable health professionals to better understand the genomes of viruses and achieve different purposes (e.g., disease tracking and surveillance, discovery of novel pathogens, etc.). To be specific, the major advantage of PCR amplicon‐based methods is that most sequence reads generated by the NGS platform are specific to the target pathogen, which provides comprehensive coverage of the viral genomes (especially for viruses with small genomes).^28^ These methods have previously been used to track the Ebola epidemics and understand the transmission event of norovirus in various settings (e.g., community).^29^, ^30^ The target enrichment methods (e.g., hybrid capture‐based target enrichment) offer the advantage of being able to sequence full viral genomes directly from clinical specimens without the need for prior culture or PCR amplification.^26^ These methods generally depend upon the available reference sequences for the virus(es) of interest. When designing probes for larger panels of reference sequences, these methods can help better capture the diversity of the target virus genomes and analyse viral populations, whereas metagenomic sequencing is unable to do so.^28^ However, novel pathogen discovery without requiring prior knowledge of the viral genomes is the chief advantage of unbiased metagenomic sequencing methods rather than other sequencing methods. In the past, these methods have been successful in detecting novel pathogens (e.g., novel bunyavirus, and novel human papillomavirus) in samples from patients with fever, which conventional diagnostic methods had not detected.^31^, ^32^ With the introduction of these advanced sequencing technologies and methods, viral genome sequencing has become increasingly important in recent years, especially in clinical research and epidemiology.

Recent reviews have elaborated on the progress, application, and considerations of NGS technologies in infectious diseases,^25^, ^33^, ^34^, ^35^ but there is a lack of discussion on the application of NGS technologies to direct respiratory virus detection and characterisation in clinical samples. Therefore, the objectives of this review were to (i) describe the current NGS platforms employed for respiratory virus identification from clinical samples (ii) demonstrate the contribution of NGS technologies (combined with different sequencing methods) to the detection of respiratory viruses in past studies and (iii) discuss the potential implementation of NGS technologies in the clinical setting, covering the major challenges that still need to be overcome, such as bioinformatics analysis resources, and ethical concerns.

## 2 METHODS

### 2.1 Study design

This literature review was conducted according to the Preferred Reporting Items for Systematic Reviews and Protocol Meta‐Analyses (PRISMA).^36^ Ethical approval was not required in the study because there were no animal or human specimens or subjects involved.

### 2.2 Search methods for selection of studies

Searches were performed of PubMed, ProQuest, Web of Science, Scopus, and LitCovid databases for published papers from 1 January 2010 to 1 February 2021. The search strategy adopted for each database was similar and was developed using Boolean logic to combine keywords described in Table 1. The search string was developed for PubMed and then adapted for other databases (see details in Supplementary Material). We limited the search to studies published in English. EndNote X9 software was utilised for compiling articles. After removed duplicates, the screening of the title/abstract was conducted. Full‐text screening of all selected titles/abstracts was then performed based on predefined inclusion and exclusion criteria. In addition, the list of references for each full‐text article was further used to locate the missing paper that met the inclusion criteria.

**TABLE 1:** Columns A, B, and C indicate interchangeable terms combined using AND/OR

Column A	Column B	Column C
Respiratory viruses	Next‐generation sequencing	Clinical laboratory
Influenza	High‐throughput sequencing	Diagnostic laboratory
Coronaviruses		Clinical samples
Parainfluenza		
Adenovirus		
Human metapneumovirus		
Human bocavirus		
Human rhinovirus		
Respiratory syncytial virus		

*Note*: Example search term: (respiratory viruses) AND (next‐generation sequencing) AND (clinical laboratory).

### 2.3 Inclusion and exclusion criteria

We included all articles that described the use of NGS platforms in testing clinical samples. These samples were collected from patients who had previously been clinically diagnosed with respiratory diseases or highly suspected of having relevant clinical symptoms as described by the authors. Additionally, the included studies were required to detect at least one of eight common respiratory viruses (e.g., IFV, CoV, PIV, AdV, human metapneumovirus [HMPV], human bocavirus [HBoV], HRV, and RSV) by NGS technology.

Studies were excluded if (a) NGS technology was not implemented in the study, (b) samples were collected from sources other than patients (e.g., animals or environment), (c) detection of viruses other than respiratory viruses, (d) article was only available as abstract, (e) studies published in languages other than English (Table 2). In addition, we excluded reviews, editorials, news, viewpoints, and perspective articles.

**TABLE 2:** Inclusion and exclusion screening criteria

Inclusion criteria	Exclusion criteria
The use of NGS platform	The NGS was not performed
Relevant to respiratory viruses (IV, CoV, PIV, AdV, HMPV, HBoV, HRV, RSV)	Non‐human samples (e.g., animal or environment samples)
Related to respiratory virus infections	Not related to detection of respiratory viruses
Human clinical samples	Studies available as abstract only
Studies published in English	Studies published in language other than English
Paper published in the search date range^a^	Review/Viewpoint/Editorial/News/Perspective publications
Full‐text articles	

Abbreviations: AdV, adenovirus; CoV, coronaviruses; HBoV, human bocavirus; HMPV, human metapneumovirus; HRV, human rhinovirus; IV, influenza virus; PIV, parainfluenza virus; RSV, respiratory syncytial virus.

^a^ Paper published from 1 January 2010, to 1 February 2021.

## 3 RESULTS

### 3.1 Identification of studies

**FIGURE 1:**
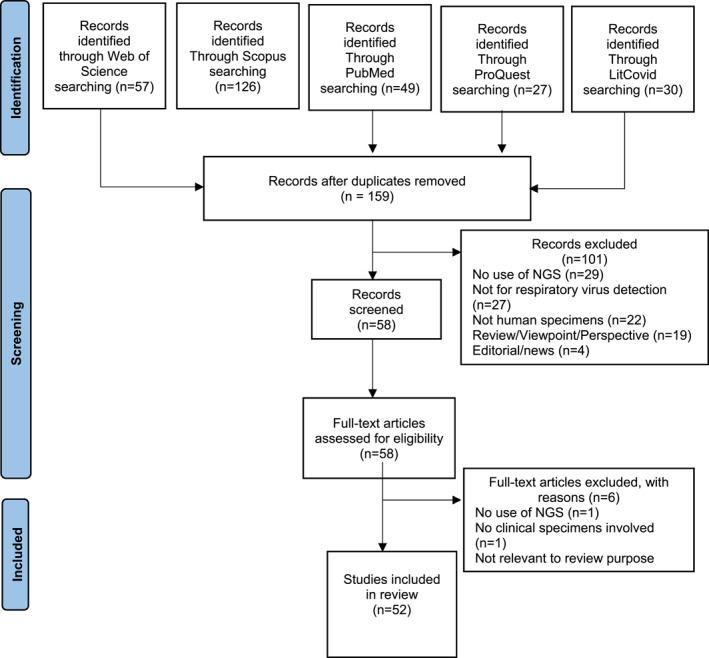
PRISMA flow diagram of literature search and selection process

Comprehensive search yielded 289 articles from five different databases (Figure 1). Of these, 159/289 records remained after removing duplicates, and 101/159 articles were excluded after screening titles and abstracts. The remaining 58 publications were then selected for full‐text review. After a full evaluation, we excluded 6 out of 58 studies not meeting the predefined inclusion criteria. There were 52 publications that met the inclusion criteria and were included in this review as summarised in Table S2 (see Supplementary Material).

### 3.2 Characteristics of included studies

Included publications described studies conducted across 18 countries with 44% (*n* = 23) of studies clustered in Asia, 29% (*n* = 15) in Europe, 17% (*n* = 9) in North America, 8% (*n* = 4) in South America, and 2% (*n* = 1) in Africa. Although the studies were published between 2011 and 2021, there was a significant increase in publications since 2019 (Supplementary Figure S1). This may be due to the increased demand from researchers and medical professionals for NGS technologies during the coronavirus disease 2019 (COVID‐19) pandemic. In addition, the included studies indicated that the NGS technology can be successfully used to detect potential known and unknown respiratory viruses from patients' clinical specimens and provide more detailed typing information. Among the respiratory viruses, SARS‐CoV‐2 (*n* = 21, 40%) and influenza virus A/B (*n* = 9, 17%) were the most frequently studied viruses by authors in included studies.

The clinical specimens selected for NGS in included studies were upper respiratory specimens, lower respiratory specimens, and faecal specimens. The included studies used Illumina sequencers (*n* = 35, 67%) most commonly, followed by Oxford Nanopore sequencers (*n* = 8, 15%), Life Technologies sequencers (*n* = 5, 10%), Roche sequencers (*n* = 3, 6%), and PacBio sequencer (*n* = 1, 2%). The specific models of the NGS platforms involved in studies are listed in Table S3 (see Supplementary Material S1). Besides, the summary of the main characteristics of all included studies are described in Table 3. Detailed information on each individual study is provided in Table S2 (see Supplementary Material).

**TABLE 3:** Characteristics of the 52 included studies

Characteristics	*N* (%)
Study population	
Children	11 (21%)
Adults	7 (13%)
Mixed	3 (6%)
Not reported	31 (60%)
Specimen type	
Upper respiratory specimens	31 (60%)
Lower respiratory specimens	2 (4%)
Mixed (the above two)	15 (30%)
Fecal specimens	1 (2%)
Not reported	2 (4%)
Study location	
Asia	23 (44%)
Europe	15 (29%)
North America	9 (17%)
South America	4 (8%)
Africa	1 (2%)
Virus detection	
SARS‐CoV‐2	21 (40%)
Influenza A/B virus	9 (17%)
Parainfluenza virus	3 (6%)
Human metapneumovirus	2 (4%)
Respiratory syncytial virus	1 (2%)
Human coronavirus (OC43)	1 (2%)
Mixed respiratory viruses^a^	15 (29%)
NGS platform^b^	
Roche	3 (6%)
Illumina	33 (63%)
Life technologies	5 (10%)
Oxford nanopore technologies	8 (15%)
Pacific BioSciences	1 (2%)
Unclear	3 (6%)

^a^ Mixed respiratory viruses include IV/HCoV/PIV/AdV/HMPV/HBoV/HRV/RSV.

^b^ Some of included studies used one or more NGS platforms.

Three sequencing methods based on NGS technologies were used to sequence respiratory viruses' genomes, including PCR amplicon sequencing (*n* = 20, 38%), metagenomic sequencing (*n* = 26, 50%), and target enrichment sequencing (*n* = 5, 9%). Among these included studies, none has used NGS technologies directly to diagnose respiratory viruses. There can be several explanations for this phenomenon, such as high sequencing costs, ethical concerns (e.g., accidently discovering the genes of other pathogens than pathogens, or accidently discovering host genetics related to underlying diseases), or regulatory issues (e.g., standards establishment).^28^, ^37^ Though there are several obstacles to the clinical application of NGS, two publications have highlighted the advantages of NGS over conventional diagnostic methods (e.g., diagnostic RT‐PCR and virus culture) in the performance of providing virus typing and serotyping, particularly for viruses with atypical subtypes (e.g., H7N9 and H10N8 of IFAs).^38^, ^39^ Furthermore, 29 studies (55.8%) have shown that NGS technologies are currently used to detect respiratory viruses in clinical samples primarily for research purposes in virology or pathology laboratories. Whereas four studies (9.6%) performed NGS in clinical microbiology laboratories, demonstrating the potential of NGS to monitor outbreaks in clinical settings^40^, ^41^ and enhance clinical diagnosis in conjunction with other traditional diagnostic tests (e.g., PCR).^42^, ^43^

### 3.3 Contribution of NGS technologies in detection of respiratory viruses

#### 3.3.1 Influenzaviruses type A and B

Eighteen studies (34.6%) in this review documented the role of NGS technologies in virus typing and mutation detection of IFVs. Among these studies, nine studies were exclusively targeting influenza viruses, and the other nine studies focussed on detecting a viral panel include influenza viruses and other respiratory viruses. The subtypes detected in these studies include influenza A H1N1pdm2009, H3N2, H7N9, H10N8, and influenza B Yamagata lineage. Five out of 18 studies (27.8%) reported the successful detection of mutations in influenza viruses through the WGS using the NGS technique.^14^, ^38^, ^44^, ^45^, ^46^ For example, Piralla et al. detected 222G/N/A mutations in the HA gene of H1N1pdm09 strains in 30% of low respiratory tract samples from patients in intensive care units. These mutations were shown to replicate more effectively in the lower respiratory tract and be more likely to increase the severity of the disease.^14^ Nieto et al. observed the D701N mutation of polymerase basic protein 2 (PB2) segment of the H1N1pdm09 virus in one patient with severe influenza.^46^ The study provided evidence on the association between the PB2‐D701N mutation and viral pathogenicity in humans and suggested that this mutation may result in more severe clinical outcomes of human infections.

In addition to providing information on virus typing and amino acid changes, NGS methods can also be considered the complement to traditional infection prevention and control (IPC) methods to uncover the putative transmission between infected patients and track the potential spread. For example, Roy et al. found that the whole genome analysis based on the NGS platform not only accurately identified the previously known influenza outbreak in the hospital, but also identified a cluster of two infections that had been previously missed by the conventional IPC method (based on HA and NA sequencing).^47^ Moreover, Roy and colleagues showed that the phylogenetic and pairwise distance analysis of WGS could assist in refuting suspicions of transmission in the haemato‐oncology wards detected by the IPC data. All these findings demonstrated the effectiveness of the NGS‐based method in outbreak surveillance and the potential for routine use of NGS technologies in clinical settings.

The NGS platforms used in the 18 studies were Illumina sequencers (MiSeq/GA II/HiSeq/NextSeq) (*n* = 14, 77.8%), Life Technologies' sequencers (Ion Torrent PGM) (*n* = 3, 17%), Oxford Nanopore's sequencers (MinION) (*n* = 2, 11.1%), and Roche's sequencers (454 GS system) (*n* = 3, 16.7%).

Furthermore, two non‐sequence‐specific viral enrichment strategies were mentioned by five studies to increase the proportion of viral sequence obtained by NGS and increase the specificity of NGS. The first strategy was to employ ultracentrifugation, sample filtration, or nuclease‐treatment method to enrich viral particles before the nucleic acid extraction step.^44^, ^48^, ^49^, ^50^, ^51^ One study attempted to utilise combinations of filtration and nuclease treatment to obtain higher genome sequence coverage and enough reads to make a single nucleotide polymorphism (SNP) analysis of the H7N9 virus.^44^ In contrast, another study selected the centrifugation approach instead of the filtration approach to increase the proportion of viral reads (H3N2 virus) derived from clinical samples because of its low cost and easy handling.^49^ In addition, one study adopted the combination methods of centrifugation and nuclease treatment to digest cellular nucleic acids in an attempt to obtain higher genome coverage.^51^ All these studies show that a pretreatment step before nucleic acid extraction can be beneficial for enhancing virus detection. However, the choice of a specific pretreatment method (e.g., ultracentrifugation, sample filtration, and/or nuclease‐treatment) depends on considerations such as cost and operational difficulty.^51^ The second strategy was to select the appropriate nucleic acid extraction kit to enable more sensitive identification of the RNA virus genome in clinical samples by NGS. One study conducted to evaluate the impact of four extraction kits in NGS analysis^52^ concluded that the Qiagen extraction kit (RNeasy Plus Micro Kit) was the most applicable for metagenomic analysis of viral RNA and enabled more sensitive identification of the viral RNA genome in clinical respiratory samples.^52^

#### 3.3.2 Severe acute respiratory syndrome coronavirus 2 (SARS‐CoV‐2)

Thirty‐one studies (59.6%) in this review documented the use of NGS techniques in coronavirus‐related studies. Among these studies, 21 studies (67.7%) were focussed on SARS‐CoV‐2, and the other 10 studies (32.3%) were related to a viral panel including common human coronaviruses (HCoV‐229E, HCoV‐HKU1, HCoV‐NL63, and HCoV‐OC43) and other respiratory viruses. The included studies all showed that NGS technologies combining different sequencing methods have been successfully used for novel coronavirus detection, virus typing, and mutation detection in this COVID‐19 pandemic. The most used sequencing method in prior studies was the PCR amplicon sequencing method, which helped researchers understand the molecular epidemiology and genetic variability of SARS‐CoV‐2 genomes in different regions. Five studies showed that NGS allowed the identification of amino acid mutation D614G in the SARS‐CoV‐2 spike protein from clinical specimens.^53^, ^54^, ^55^, ^56^, ^57^, ^58^ Moreover, three studies conducted during the COVID‐19 pandemic emphasised the superiority of the application of NGS over current diagnostic tools (e.g., RT‐PCR or qRT‐PCR) in tracing chains of transmission.^56^, ^58^, ^59^ Phylogenetic analyses based on genomic sequence data generated by NGS in these studies revealed the importation or local transmission chains that were not detected by traditional contact tracing strategies or travel history.

NGS platforms used in these 31 studies included Illumina (MiSeq/HiSeq X/HiSeq 2500/MiniSeq/NovaSeq/NextSeq/iSeq) sequencers (*n* = 21, 68%), Life Technologies' (Ion Torrent) sequencers (*n* = 1, 3%), Oxford Nanopore (MinION) sequencers (*n* = 6, 19%), and Roche (454 GS junior) sequencer (*n* = 1, 3%). Five studies used two NGS platforms in the research.^55^, ^56^, ^59^, ^60^, ^61^

Two target enrichment sequencing strategies used for SARS‐CoV‐2 detection were used in two studies. The first strategy was the use of the two‐stage metagenomic RNA enrichment viral sequencing (MINERVA) protocol. This protocol was shown to be applicable to various clinical specimens to obtain high genome coverage of SARS‐CoV‐2.^62^ Meanwhile, compared with the existing RNA sequencing methods, this protocol shortened the hands‐on operation time and required less expert technique, which might be easier for clinical personnel to conduct in future outbreaks. Another strategy was to utilise the Agilent SureSelect^XT^ target enrichment system.^63^ O'Flaherty and colleagues (2018) observed 50%–99% of the reads per sample were the target viral reads after enrichment using this strategy, compared to only 0.3% of targeted viral reads per sample without the strategy. Their results highlighted the effectiveness of using the Agilent SureSelect^XT^ target enrichment system in respiratory virus detection and provided information for the selection of target enrichment methods for similar studies in the future.

Importantly, NGS results can be used in synergy with other diagnostic test results to enhance the final clinical diagnosis of patients, as in three SARS‐CoV‐2 studies.^64^, ^65^, ^66^ Take one study as an example,^65^ at the beginning of the COVID‐19 outbreak, four SARS‐CoV‐2 real‐time reverse transcription‐PCR (rRT‐PCR) tests were performed on the upper respiratory tract specimens (including nasopharyngeal swab specimens and sputum specimen) of a suspected COVID‐19 patient by the health professionals. However, none of these rRT‐PCR results were positive. Subsequently, Wu and his colleagues collected another nasopharyngeal swab to detect SARS‐CoV‐2 and used the Xpert Flu/RSV Xpress assay to differentiate influenza A and B from respiratory syncytial viruses in the same sample. This sample was detected as influenza A positive, but SARS‐CoV‐2 negative. Considering the patient's travel history to Wuhan, Wu and his colleagues then decided to use mNGS method to detect the unknown pathogens from the patient's lower respiratory tract sample (bronchoalveolar lavage fluid (BALF)). The sequences generated by mNGS showed 99.8% identity, covering approximately 99% of the SARS‐CoV‐2 (Wuhan‐Hu‐1) genome (GenBank accession number NC_045512.2). This finding was then confirmed by rRT‐PCR for SARS‐CoV‐2 on the same BALF sample to eventually confirm the diagnosis of the patient and provided helpful information for clinicians to design the corresponding clinical management. The present case illustrates that COVID‐19 may go undiagnosed due to false‐negative rRT‐PCR results in the upper respiratory tract samples or co‐infection with other respiratory viruses. In this context, unbiased mNGS proved to be a useful tool to complement existing laboratory methods, providing the near‐full‐length SARS‐COV‐2 genome to help medical professionals make diagnostic decisions. As well, this case illustrates the capability of metagenomic sequencing to detect SARS‐CoV‐2 in a challenging situation where influenza A co‐infection is present.

#### 3.3.3 Other respiratory viruses

Studies have documented that the metagenomic sequencing method based on NGS technologies usually can be used to detect multiple respiratory viruses simultaneously from clinical samples.^16^, ^37^, ^48^, ^50^, ^63^, ^67^, ^68^, ^69^, ^70^, ^71^, ^72^, ^73^, ^74^, ^75^, ^76^ These include RSV, HPIV, HAdV, HRV, HMPV, and HBoV. Researchers can obtain partial or even complete genome sequences of each above virus and provide detailed subtyping information by using the NGS technologies. Moreover, these studies targeting the detection of a panel of different viruses in a single experiment have also highlighted the time‐saving and labour‐saving benefits of NGS techniques compared with traditional methods.^37^, ^75^, ^77^ In addition, the RNA sequencing (RNASeq) method based on the NGS technique was conducted in a study not only assisted researchers in identifying the RSV genome through de novo assembly but also helped researchers understand the genetic variability of RSV.^67^ For HPIV research, metagenomic sequencing methods allowed researchers to detect a divergent HPIV type 4^68^ and uncover the transmission chains in the small outbreak of hospital‐acquired HPIV type 3 infections.^16^ Second‐generation sequencing platforms (especially Illumina sequencers) continued to be the primary platforms of researchers' choices in above studies.

### 3.4 Validation of NGS results

Previous studies suggest several methods that can be used to validate NGS results to ensure the results' accuracy. Six out of 52 (11.5%) studies used Sanger's sequencing methods to confirm the NGS results.^16^, ^37^, ^38^, ^44^, ^50^, ^77^ Three studies (5.8%) performed real‐time quantitative reverse transcription PCR (qRT‐PCR) assays on the same specimens to confirm the NGS results.^39^, ^73^, ^78^ Two studies (3.8%) attempted to perform two NGS platforms on same specimens and use the result of one platform to validate the result of the other.^49^, ^79^

### 3.5 Financial costs of performing NGS

Five studies (9.6%) showed that the cost of conducting NGS in the laboratory varied depending on the selection of NGS platforms, the choice of library preparation kits, the selection of sequencing methods, as well as the associated NGS‐based experimental purposes.^16^, ^40^, ^78^, ^80^, ^81^ For example, the instrument cost and sequencing cost vary according to the specific model of NGS platforms. Oxford Nanopore MinION sequencer has received a lot of labs' attentions in recent years, primarily because of its low initial investment compared to other NGS platforms with high instrument costs. MinION sequencers require no capital investment and only a minimal supporting laboratory infrastructure, which may potentially reduce the total sequencing cost.^55^ In addition, different sequencing methods will also lead to various sequencing costs. For example, when compared to the ARTIC amplicon protocol and sequence capture methods, the tailed amplicon method developed by Gohl et al. was shown to reduce the sequencing cost to $20–40 per sample, which may enable large‐scale genomic surveillance of SARS‐CoV‐2 in the future.^80^ Whereas the metagenomic sequencing method shown in another included study was approximately $75 per sample, which was significantly higher than the tailed amplicon method mentioned above.^81^

### 3.6 Barriers to the routine use of NGS in clinical laboratories

Although past studies have demonstrated many benefits of NGS, authors still discussed several barriers if clinical laboratories are considering implementing NGS in routine diagnosis: (i) the cost of conducting NGS work is still relatively high compared to currently available standard diagnostic tools,^39^, ^40^, ^48^, ^49^, ^78^ (ii) the whole process of NGS work requires lengthy turnaround times and skilled laboratory staff, especially the library preparation step. Existing diagnostic tests, such as RT‐PCR or rapid antigen tests, have shorter turnaround times in providing the results and simple processing requirements,^39^, ^49^, ^78^ (iii) the high volume and complexity of sequence data require sophisticated downstream bioinformatic analyses conducted by skilled laboratory staff,^46^, ^48^, ^49^, ^78^ (iv) contamination from the host or microbiota cells may result in low viral genetic material, especially when nasal specimens were used for metagenomic sequencing,^42^, ^46^, ^55^ (v) limited quality assurance programs are currently available to validate NGS results, unless the confirmatory experiments are conducted using traditional molecular methods; standard validation methods should be established when NGS techniques are used in future diagnoses.^37^

## 4 DISCUSSION

We systematically identified 52 studies on respiratory virus identification that used NGS methods, reporting the use of different NGS platforms and the specific contribution of NGS technology in the research. It is not possible to directly compare the performance of different NGS‐based methods due to the methodological heterogeneity among studies. However, all included studies suggest that these NGS‐based methods improve upon traditional molecular techniques primarily in generating high‐throughput, increasing the quantity of sequencing data, and providing more comprehensive genomic information.

The NGS technologies have become more widely used in the field of respiratory virus research in the past decade. With the continuous improvement and development of technology, the NGS platforms have rapidly evolved from second‐generation (2G) sequencing platforms to third‐generation (3G) platforms and fourth‐generation (4G) platforms, resulting in a variety of NGS platforms available for researchers to choose. There are 14 specific modes of NGS platforms summarised from the search (See Table S2). 2G platforms from Illumina were the most selected instrument in previous studies (40/52, 76.9%), primarily because of its higher accuracy (about 99%) of sequence data with a lower error rate compared to other NGS platforms.^38^ In addition to Illumina 2G platforms, 3G and 4G platforms have gained attention in recent years due to their ability to produce longer reads to increase the quality of genome assemblies.^40^, ^41^, ^49^, ^56^, ^60^, ^61^, ^68^, ^82^, ^83^, ^84^ In particular, the Oxford Nanopore sequencers (e.g., MinION and GridION; 4G platform) have been increasingly used for virus detection during the COVID‐19 pandemic. The additional advantages of these Nanopore sequencers include their portability in field studies, low initial installation costs, and simple library preparation procedure with the minimum laboratory equipment requirements.^40^, ^56^, ^61^, ^83^, ^84^, ^85^ But the main concern of such sequencers is to have a relatively higher error rate (approximately 3%–12%) than other 2G platforms (e.g., Illumina's error rate <0.01%).^40^ Therefore, the results generated by such sequencers need to be carefully interpreted. These above findings also suggest that if clinical laboratories are to implement high‐quality NGS work in the future, lab personnel should consider the following factors before selecting the appropriate NGS platform, including the sequencing costs, requirements on laboratory infrastructure, run time, analysis time, error rate, and convenience.

Furthermore, included studies suggest that different sequencing methods can assist researchers in achieving different purposes, in addition to selecting an appropriate NGS platform. In light of the studies reviewed, researchers usually employed the PCR amplicon sequencing method when they performed the evolutionary or epidemiological analysis of one target respiratory virus, especially during outbreaks.^53^, ^80^, ^86^ This method could enable researchers to analyse the genomes of epidemic‐prone viruses in a quick way and detect mutations/variants, which may be beneficial for future drug and vaccine improvement or development. Aside from that, researchers also utilised the PCR amplicon WGS method when they attempted to reveal transmission events of the virus (e.g., IFVs and SARS‐CoV‐2).^56^ Nevertheless, when researchers sought to identify unknown or novel respiratory pathogens in patients who were suffering from unknown febrile illnesses or had co‐infection with different respiratory viruses, metagenomic sequencing methods were always the preferred method rather than any other sequencing methods.^64^ Another situation where metagenomics sequencing was considered was when researchers did not detect any viruses in patients with respiratory symptoms using the routine culture and PCR methods.^48^, ^74^, ^77^ Moreover, the target enrichment sequencing method was not commonly used in our included studies, yet it can be a viable option for identifying multiple known respiratory viruses or co‐infections from samples (conventional laboratory methods might fail to detect).^63^ The information provided here can be considered as a suggestion for clinical laboratories when they select sequencing methods based on NGS technologies for respiratory virus monitoring or diagnosis in the future.

To date, in addition to the aforementioned barriers to limiting the routine use of next‐generation sequencing in clinical laboratories (e.g., high sequencing costs, complex bioinformatics data analysis, the requirement of skilled laboratory staff, etc.), other three major challenges were also mentioned by previous reports that may slow the adoption of NGS in clinical practice.^28^, ^87^, ^88^, ^89^ The first major challenge is the potential ethical issues that may arise during NGS. For example, if HIV infection is detected in the patient during metagenomic sequencing for respiratory pathogens, should health professionals notify the patient or not. Or incidental finding of other diseases rather than the original investigation purpose. How to handle this situation and minimise the potential harm to the patient will be important for future studies to carefully consider. In the above scenarios, although amplicon and target enrichment sequencing methods have the advantage of providing only virus‐related results, which allows them to avoid these ethical problems, other ethical issues may arise when these methods are used to provide results related to disease transmission events. For example, when who infects whom during the outbreak investigation is identified, how to protect the confidentiality of patients will be another issue to consider. The second challenge could be storing genomic data in the clinical setting. NGS technologies generally generate substantial amounts of genomic data. Who has access to the genomic data, and how genomic data is stored and maintained should be also discussed in the future. The third challenge is how to interpret NGS findings appropriately. For example, different sequences of pathogens can be detected during mNGS, but not all of these sequences will contribute to the patient's current disease. How NGS results are interpreted in terms of clinical symptoms and previous records will also be critical to diagnosis. As a result, current diagnostic tests (such as NAATs) cannot be replaced by NGS methods unless all of the above obstacles are overcome.

NGS technologies were proven that had a broad prospect in diagnosing respiratory tract infection, identifying nosocomial cross‐infection, drug resistance research, and other aspects. More than that, when faced with potential emerging infectious diseases (e.g., the current COVID‐19 pandemic), NGS technologies enable us to gain a deeper and easier understanding of the genome of the virus, understand its pathogenesis and develop more effective vaccines against it. Therefore, if NGS is to be introduced into routine clinical diagnosis, future research should be more focussed on addressing the above barriers, exploring how to improve the specificity and sensitivity of NGS technology in diagnosis, and how to further optimise and standardise NGS experiments in clinical laboratories.

## 5 CONCLUSIONS

The advent of NGS technology has revolutionised the way we understand viral genomes over the past decade. Although NGS technology has been widely used to diagnose hereditary disorders and advance the personalised treatment of cancer in clinical laboratories,^90^, ^91^ there is still some way to go before the NGS is used to diagnose viral respiratory tract infections. The rapid diffusion of NGS into clinical laboratories for detecting respiratory viruses will be based on whether the challenges relating to NGS can be addressed.^39^, ^42^, ^46^, ^48^, ^49^, ^78^ Notably, the cost of NGS has been reduced over time, which will attract more laboratories to bring this technique into their research and make efforts in optimising NGS protocols. Moreover, a few commercial software packages, such as Geneious and Qiagen CLC Workbench, offer an easy‐to‐use graphical interface for analysing complex NGS data, which may be beneficial to those without bioinformatics training. It can be expected that the rapid development of NGS technologies and bioinformatics pipelines may address the current challenges of NGS platforms in the future. Thus, we can expect that NGS technology will be widely applied in clinical laboratories for routine practice at an affordable cost and play an essential role in diagnosing respiratory diseases, developing more appropriate treatment, and surveilling emerging viruses in the near future.

## AUTHOR CONTRIBUTIONS

**Xinye Wang:** designed the review, searched, screened, and extracted literature, and wrote the manuscript. Matthew Scotch and Sacha **Stelzer‐Braid:** guided on the searching method, reviewed the searching results, and edited the manuscript. **William D. Rawlinson:** guided, reviewed, and edited the manuscript. All authors reviewed and approved the manuscript before submission.

## CONFLICT OF INTEREST

The authors declare that they have no conflicts of interest.

## Supporting information

Supporting Information S1

## Data Availability

The data used to support the findings of this study are included within this manuscript.
