# Supplementary material for: Detection of respiratory viruses directly from clinical samples using next‐generation sequencing: A literature review of recent advances and potential for routine clinical use
Source: Rev Med Virol. 2022 Jul 1;32(5):e2375. doi: 10.1002/rmv.2375 (PMC9539958; doi:10.1002/rmv.2375)
Supplement: Supplementary file 1 — Supporting Information S1 [file RMV-32-e2375-s001.docx]

**Supplementary Material**

**Detection of Respiratory Viruses Directly from Clinical Samples Using Next-Generation Sequencing: A Literature Review of Recent Advances and Potential for Routine Clinical Use**

Xinye Wang^1,2^, Sacha Stelzer-Braid^1,2^, Matthew Scotch^3,4^, William D. Rawlinson^1,2*^

**Affiliations:**

^1^ Virology Research Laboratory, Serology and Virology Division (SAViD), NSW Health Pathology, Prince of Wales Hospital, University of New South Wales, Sydney, Australia

^2^ School of Medical Sciences, Faculty of Medicine, University of New South Wales, Sydney, NSW, Australia

^3^ Kirby Institute, University of New South Wales, Sydney, NSW, Australia

^4^ Biodesign Center for Environmental Health Engineering, Biodesign Institute, Arizona State University, Tempe, Arizona, USA

**Introduction**

**Table S1. Advantages and Disadvantages of Various Laboratory Methods for Respiratory Virus Detection**

| **Laboratory Method** | **Advantages** | **Disadvantages/Limitations** |
| --- | --- | --- |
| Virus Isolation | Direct measure of infectivity | Expensive, labour-intensive, and time-consuming |
|  | Further material can be produced for the study of agent | May require special facilities (e.g., BSL-3 lab) |
|  | Highly sensitive | Inability to provide virus typing information |
|  |  | Inability to detect unknown pathogens |
| DFA | Simple to use | Subjectiveness (requires the technical expertise for accurate interpretation) |
|  | Rapid test results | Labour-intensive and time-consuming evaluation |
|  | Inexpensive | Inability to provide virus typing information |
|  |  | Inability to detect unknown pathogens |
| RADTs | Simple to use | Moderate sensitivity |
|  | Rapid test results | False negatives results |
|  | Inexpensive | Qualified personnel |
|  | High specificity | Inability to provide virus typing information |
|  | Can be performed at point-of-care | Inability to detect unknown pathogens |
| NAATs (e.g., PCR) | Superior sensitivity, and high specificity | Higher cost |
|  | Multiplexing is possible | Risk of cross-contamination |
|  | Requires less time for the detection | Target specific agents |
|  | No requirement for calibration | Limited to provide antibiotic resistance data and virus typing information |
|  |  | Primer’s design is important, which may affect the result |
| NGS | Ability to simultaneously sequence millions of small DNA fragments in parallel | Higher cost |
|  | Ability to generate a large yield of sequence data at high speed | Require for lengthy turnaround times and skilled laboratory staff during the process of NGS work |
|  | Ability to provide more comprehensive genomic information (e.g., virus typing) | Skilled laboratory staff is required for conducting sophisticated downstream bioinformatic analyses |
|  | Ability to detect known and unknown pathogens without prior knowledge |  |
|  | Higher sensitivity to detect low-frequency variants |  |

Abbreviations: DFA (Direct fluorescent antibody testing; RADTs (Rapid Antigen Direct Tests); NAATs (nucleic acid amplification tests); NGS (next-generation sequencing)

**Methods**

Search formulae of PubMed was listed as below:

#1 ("respiratory virus*" OR "respiratory pathogen" OR influenza OR parainfluenza OR adenovirus* OR coronavirus* OR "human metapneumovirus*" OR "human bocavirus*" OR "respiratory syncytial virus*" OR "human rhinovirus*)

#2 ("next-generation sequenc*" OR "high throughput sequenc*")

#3 ("diagnostic laborator*" OR "clinical laborator*" OR "clinical sample*")

#4 #1 AND #2 AND #3

Similar search string was used for Web of Science, Scopus, ProQuest, and LitCovid.

**Results**

**Table S2.** Characteristics of the included studies (n=52)

| **Author Name** | **Country** | **Publication Year** | **Virus Detected** | **Study population** | **Clinical samples** | **NGS Platform** |
| --- | --- | --- | --- | --- | --- | --- |
| Ai J-W, et al ^1^ | China | 2020 | SARS-CoV-2 | Adult | Pharyngeal swab | Illumina Nextseq |
| Aljabr W, et al ^2^ | UK | 2019 | RSV | Children | Nasopharyngeal aspirates | Illumina HiSeq 2000 |
| Alquezar-Planas DE, et al ^3^ | Denmark | 2013 | HPIV | Children | Respiratory secretions | 454 GS FLX Titanium/PacBio SMRT RS II |
| Alvarez-Diaz DA, et al ^4^ | Colombia | 2020 | SARS-CoV-2 | No reported | Nasopharyngeal swabs | Oxford Nanopore MinION/Illumina MiSeq |
| Bal A, et al ^5^ | France | 2018 | RSV, HCoV-(NL63/229E), HMPV, IFV, HBoV, HAdV, HPIV | No reported | Nasopharyngeal swabs, aspirates, or sputums | Illumina NextSeq 500 |
| Bartolini B, et al ^6^ | Italy | 2020 | SARS-CoV-2 | Adults | Nasopharyngeal swabs, bronchoalveolar lavage samples | Life Technologies Ion Torrent |
| Bhoyar RC, et al ^7^ | India | 2021 | SARS-CoV-2 | No reported | Nasal, nasopharyngeal, oropharyngeal swabs | Illumina NovaSeq 6000 |
| Carbo EC, et al ^8^ | Netherland | 2020 | SARS-CoV-2 | No reported | Nasopharyngeal swabs | Illumina NovaSeq 6000 |
| Chan JF, et al ^9^ | China | 2020 | SARS-CoV-2 | Children and Adults | Nasopharyngeal swabs, sputums | Oxford Nanopore MinION |
| Chen C, et al ^10^ | China | 2020 | SARS-CoV-2 | Adults | Pharyngeal swabs, sputums | Illumina NextSeq 500 |
| Foster MW, et al ^11^ | Canada | 2015 | HMPV | Children | Nasopharyngeal aspirates | Illumina MiSeq |
| Gohl DM, et al ^12^ | USA | 2020 | SARS-CoV-2 | No reported | Non-specific clinical biospecimens | Illumina MiSeq |
| Gong YN, et al ^13^ | Taiwan, China | 2016 | RSV, HAdV | No reported | Nasopharyngeal, throat swabs | Illumina system (non-specific) |
| Gong YN, et al ^14^ | Taiwan, China | 2017 | HRV, HPIV, CoV, RSV, HMPV | Children | Throat swabs, nasopharyngeal swabs, and sputums | Illumina MiSeq |
| Graf EH, et al ^15^ | USA | 2016 | HMPV, AdV, IFV, PIV, HRV, RSV | Children | Nasopharyngeal swabs | Illumina HiSeq 2500 |
| Greninger AL, et al ^16^ | USA | 2017 | PIV | Children | Nasal swabs | Illumina MiSeq |
| Hourdel V, et al ^17^ | France | 2020 | SARS-CoV-2 | No reported | Sputums | Oxford Nanopore MinION/Illumina iSeq100 |
| Hu X, et al ^18^ | China | 2020 | SARS-CoV-2 | Adults | Nasopharyngeal swabs, sputums | Illumina NovaSeq |
| Hu Y, et al ^19^ | China | 2015 | IFV (H7N9) | No reported | Tracheal aspirate specimen | Life Technologies Ion Torrent PGM |
| Iketani S, et al ^20^ | USA | 2020 | HPIV | Children | Nasal swabs | Illumina MiSeq |
| Imai K, et al ^21^ | Japan | 2018 | IFV | No reported | Nasopharyngeal swabs | Oxford Nanopore MinION/Illumina MiSeq |
| Isa P, et al ^22^ | Mexico | 2019 | IFV | No reported | Nasopharyngeal swabs | Illumina Genome Analyzer II |
| Jazaeri Farsani SM, et al ^23^ | Netherland | 2015 | HCoV(OC43), IFV | No reported | Nasal swabs | Roche 454 GS Jinior System |
| Kamau E, et al ^24^ | Kenya and Zambia | 2020 | HMPV | Children | Nasopharyngeal , oropharyngea swabs | Illumina MiSeq |
| Klempt P, et al ^25^ | Czech Republic | 2020 | SARS-CoV-2 | No reported | Nasopharyngeal swabs, bronchoalveolar lavage fluid | Illumina MiSeq |
| Kustin T, et al ^26^ | Israel | 2019 | HAdV, IFV, RSV, PIV | No reported | Nose throat swab | Illumina MiSeq |
| Laiton-Donato K, et al ^27^ | Colombia | 2020 | SARS-CoV-2 | No reported | Nasopharyngeal swabs | Oxford Nanopore MinION/Illumina MiSeq |
| Lewandowski K, et al ^28^ | UK | 2019 | IFV | No reported | Throat and nose swabs | Oxford Nanopore MinION/Illumina MiSeq |
| Lu J, et al ^29^ | China | 2020 | SARS-CoV-2 | Children and Adults | Nasopharyngeal, throat, anal swabs | Oxford Nanopore MinION |
| Madi N, et al ^30^ | Kuwait | 2018 | IFV, RSV, HBoV, HRV, HCoV(HKU14), HMPV | Children and Adults | Nasopharyngeal swab, bronchoalveolar lavage, tracheal aspirates, sputum, throat swabs, nasal swabs | Illumina MiSeq |
| Maurier F, et al ^31^ | France | 2019 | HCoV(OC43) | No reported | Nasal swab or broncho-alveolar lavage | Illumina MiSeq |
| Moore et al ^32^ | UK | 2020 | SARS-CoV-2 | No reported | Nasopharyngeal swabs | Oxford Nanopore MinION/GridION |
| Nieto et al ^33^ | Spain | 2017 | IFV | No reported | Respiratory sample (non-specific) | Illumina HiSeq2000 |
| O'Flaherty BM, et al ^34^ | USA | 2018 | HRV, HMPV, HCoV | No reported | Nasopharyngeal swab, oropharyngeal swab, lung tissue | Illumina MiSeq |
| Parker J, et al ^35^ | USA | 2017 | IFV (H1N1pdm) | No reported | Nasopharyngeal swabs | Illumina MiSeq |
| Pérez-Sautu U, et al ^36^ | Spain | 2019 | HRV, HMPV, HCoV, RSV, IFV | Children | Nasopharyngeal aspirates | Illumina MiSeq/NextSeq 500 |
| Piralla A, et al ^37^ | Italy | 2017 | IFV (H1N1pdm) | No reported | Nasal swabs, nasopharyngeal aspirates, bronchoalveolar lavage, broncho aspirates | Roche GS Junior 454 system |
| Ren X, et al ^38^ | China | 2015 | IFV | No reported | Sputums | Life Technologies Ion Torrent PGM/Illumina MiSeq |
| Roy S, et al ^39^ | UK | 2019 | IFV | No reported | Nasopharyngeal aspirates | Illumina MiSeq |
| Sarkale P, et al ^40^ | India | 2020 | SARS-CoV-2 | No reported | Throat swab, nasal swab, oropharyngeal swab, sputum | non-specific NGS platform |
| Shah SJ, et al ^41^ | USA | 2020 | SARS-CoV-2 | No reported | Oropharyngeal, nasopharyngeal swabs | Illumina NovaSeq 6000 |
| Thi Kha Tu N, et al ^42^ | Vietnam | 2020 | HR, IFV, HCoV (OC43), RSV | No reported | Nasal-throat swabs | Illumina MiSeq |
| Thorburn F, et al ^43^ | UK | 2015 | HRV, HCoV (229E/NL63), HMPV, PIV | Adults | Nasopharyngeal swabs | Illumina MiSeq |
| Tushir S, et al ^44^ | India | 2021 | SARS-CoV-2 | No reported | Nasopharyngeal swab | Illumina HiSeq X |
| Wu X, et al ^45^ | China | 2020 | SARS-CoV-2 | Adult | Nasopharyngeal swab, sputum, BALF specimens | non-specific NGS platform |
| Xiao F, et al ^46^ | China | 2020 | SARS-CoV-2 | Adult | fecal specimens | non-specific NGS platform |
| Yadav PD, et al ^47^ | India | 2020 | SARS-CoV-2 | Non specific | Throat, nasal swabs | Illumina MiniSeq |
| Yang J, et al ^48^ | China | 2011 | RSV, HRV, IFV, PIV, AdV, HBoV | Children | Nasopharyngeal aspirates | Illumina GA II sequencer |
| Zhang D, et al ^49^ | China | 2018 | HMPV, AdV, IFV, PIV, HCoV (OC43) | Non specific | Nasopharyngeal aspirates | Illumina HiSeq2500 platform |
| Zoll J, et al ^50^ | Netherland | 2015 | HRV, HMPV, RSV | Children | Nasopharyngeal aspirates, sputums | Life Technologies IonTorrent PGM |
| Zou XH, et al ^51^ | China | 2016 | IFV | Non specific | Nasopharyngeal swabs | Life Technologies IonTorrent PGM/Illumina MiSeq |
| Zuckerman NS, et al ^52^ | Israel | 2020 | SARS-CoV-2 | Non specific | Nasopharyngeal swabs | Illumina MiSeq |

Abbreviations:

IFV: influenza virus. RSV: respiratory syncytial virus. HRV: human rhinovirus. HMPV: human metapneumovirus. HCoV: human coronavirus. PIV:parainfluenza virus. AdV: adenovirus. HBoV: human bocavirus.

**Table S3.** A List of NGS Platforms Summarized from Selected Studies

| **Platform** | **Manufacturer** | **Sequecing Chemistry** | **Clonal Amplification** | **Run Types** | **Maximum Read Length (bp)** | **Time/Run (Estimated)^a^** |
| --- | --- | --- | --- | --- | --- | --- |
| ***Second-generation sequencing technique*** | | |  |  |  |  |
| 454 GS Jinior System | Roche | Pyrosequencing - (based on SBS) | Emulsion PCR | Single end | 500 | 18h |
| 454 GS FLX Titanium | Roche | Pyrosequencing - (based on SBS) |  | Single end | 700 | 24h |
| Genome Analyzer II | Illumina | SBS | Bridge amplification | Single & paired end | 2*150 | 14d |
| HiSeq 2000 | Illumina | SBS |  | Single & paired end | 2*125 | 60-192h |
| HiSeq 2500 | Illumina | SBS |  | Single & paired end | 2*250 | 48h |
| HiSeq X | Illumina | SBS |  | Single & paired end | 2*150 | 24-84h |
| MiSeq/MiSeqDx | Illumina | SBS |  | Single & paired end | 2*300 | 4-55h |
| NextSeq 500 | Illumina | SBS |  | Single & paired end | 2*150 | 12-30h |
| NovaSeq 6000 | Illumina | SBS |  | Single & paired end | 2*150 | 13-44h |
| MiniSeq | Illumina | SBS |  | Single & paired end | 2*150 | 4-24h |
| iSeq100 | Illumina | SBS |  | Single & paired end | 2*150 | 9-17.5h |
| Ion Torrent PGM | Life Technologies | SBS | Emulsion PCR | Single end | 600 | 2-7.5h |
| ***Third-generation sequencing technique*** | | |  |  |  |  |
| PacBio SMRT RS II | Pacific BioSciences | single-molecule real-time sequencing | N/A | Single end | 60 kb | 0.5-60h |
| ***Fourth-generation sequencing technique*** | | |  |  |  |  |
| MinION/GridION | Oxford Nanopore Technologies | Single-molecule real-time sequencing incorporating nanopore technology |  | - | 200,000 | <48h |

Abbreviations: SBS (sequencing by synthesis). Notes: the specific model of NGS platform used in each individual study is described in Table. S1 in Supplementary Material.

**Figure S1.** Distribution of studies selected for full-text evaluation by publication year

**References of included studies (n=52) in alphabetical order**

1. Ai J-W, Zhang Y, Zhang H-C, Xu T, Zhang W-H. Era of molecular diagnosis for pathogen identification of unexplained pneumonia, lessons to be learned. *Emerging microbes & infections.* 2020.

2. Aljabr W, Armstrong S, Rickett NY, et al. High resolution analysis of respiratory syncytial virus infection in vivo. *Viruses.* 2019;11(10).

3. Alquezar-planas DE, Mourier T, Bruhn CAW, et al. Discovery of a divergent HPIV4 from respiratory secretions using second and third generation metagenomic sequencing. *Scientific Reports (Nature Publisher Group).* 2013;3:2468.

4. Alvarez-Diaz DA, Franco-Munoz C, Laiton-Donato K, et al. Molecular analysis of several in-house rRT-PCR protocols for SARS-CoV-2 detection in the context of genetic variability of the virus in Colombia. *Infect Genet Evol.* 2020.

5. Bal A, Pichon M, Picard C, et al. Quality control implementation for universal characterization of DNA and RNA viruses in clinical respiratory samples using single metagenomic next-generation sequencing workflow. *BMC infectious diseases.* 2018;18.

6. Bartolini B, Rueca M, Gruber CEM, et al. SARS-CoV-2 phylogenetic analysis, Lazio Region, Italy, February–March 2020. *Emerg Infect Dis.* 2020;26(8):1842-1845.

7. Bhoyar RC, Jain A, Sehgal P, et al. High throughput detection and genetic epidemiology of SARS-CoV-2 using COVIDSeq next-generation sequencing. *PLoS One.* 2021;16(2):e0247115.

8. Carbo EC, Sidorov IA, Zevenhoven-Dobbe JC, et al. Coronavirus discovery by metagenomic sequencing: a tool for pandemic preparedness. *J Clin Virol.* 2020;131.

9. Chan JFW, Yuan SF, Kok KH, et al. A familial cluster of pneumonia associated with the 2019 novel coronavirus indicating person-to-person transmission: a study of a family cluster. *Lancet (London, England).* 2020;395(10223):514-523.

10. Chen C, Li J, Di L, et al. MINERVA: A Facile Strategy for SARS-CoV-2 Whole-Genome Deep Sequencing of Clinical Samples. *Molecular Cell.* 2020;80(6):1123-1134.e1124.

11. Foster MW, Gerhardt G, Robitaille L, et al. Targeted Proteomics of Human Metapneumovirus in Clinical Samples and Viral Cultures. *Analytical Chemistry.* 2015;87(20):10247.

12. Gohl DM, Garbe J, Grady P, et al. A rapid, cost-effective tailed amplicon method for sequencing SARS-CoV-2. *BMC Genomics.* 2020;21(1):863.

13. Gong YN, Chen GW, Yang SL, Lee CJ, Shih SR, Tsao KC. A Next-Generation Sequencing Data Analysis Pipeline for Detecting Unknown Pathogens from Mixed Clinical Samples and Revealing Their Genetic Diversity. *Plos One.* 2016;11(3):19.

14. Gong Y-n, Yang S-l, Chen G-w, et al. A metagenomics study for the identification of respiratory viruses in mixed clinical specimens: an application of the iterative mapping approach. *Archives of Virology.* 2017;162(7):2003-2012.

15. Graf EH, Simmon KE, Tardif KD, et al. Unbiased Detection of Respiratory Viruses by Use of RNA Sequencing-Based Metagenomics: a Systematic Comparison to a Commercial PCR Panel. *J Clin Microbiol.* 2016;54(4):1000-1007.

16. Greninger AL, Zerr DM, Qin X, et al. Rapid Metagenomic Next-Generation Sequencing during an Investigation of Hospital-Acquired Human Parainfluenza Virus 3 Infections. *J Clin Microbiol.* 2017;55(1):177-182.

17. Hourdel V, Kwasiborski A, Balière C, et al. Rapid Genomic Characterization of SARS-CoV-2 by Direct Amplicon-Based Sequencing Through Comparison of MinION and Illumina iSeq100(TM) System. *Front Microbiol.* 2020;11:571328.

18. Hu X, Deng Q, Li J, et al. Development and Clinical Application of a Rapid and Sensitive Loop-Mediated Isothermal Amplification Test for SARS-CoV-2 Infection. *mSphere.* 2020.

19. Hu Y, Ren X, Li L, et al. Rapid genome sequencing and characterization of novel avian-origin influenza A H7N9 virus directly from clinical sample by semiconductor sequencing. *J Clin Virol.* 2015;73:84-88.

20. Iketani S, Shean RC, Ferren M, et al. Viral Entry Properties Required for Fitness in Humans Are Lost through Rapid Genomic Change during Viral Isolation. *mBio.* 2018;9(4):19.

21. Imai K, Tamura K, Tanigaki T, et al. Whole genome sequencing of influenza A and B viruses with the MinION sequencer in the clinical setting: A pilot study. *Front Microbiol.* 2018;9(NOV).

22. Isa P, Escalera M, Cobian-Guemes AG, et al. Efficient whole genome sequencing of influenza A viruses. In. Cold Spring Harbor: Cold Spring Harbor Laboratory Press; 2019.

23. Jazaeri Farsani SM, Deijs M, Dijkman R, et al. Culturing of respiratory viruses in well-differentiated pseudostratified human airway epithelium as a tool to detect unknown viruses. *Influenza and other Respiratory Viruses.* 2015;9(1):51-57.

24. Kamau E, Oketch JW, de Laurent ZR, et al. Whole genome sequencing and phylogenetic analysis of human metapneumovirus strains from Kenya and Zambia. *BMC Genomics.* 2020;21:1-10.

25. Klempt P, Brož P, Kašný M, Novotný A, Kvapilová K, Kvapil P. Performance of Targeted Library Preparation Solutions for SARS-CoV-2 Whole Genome Analysis. *Diagnostics.* 2020;10(10):769.

26. Kustin T, Ling G, Sharabi S, et al. A method to identify respiratory virus infections in clinical samples using next-generation sequencing. *Scientific Reports (Nature Publisher Group).* 2019;9(1).

27. Laiton-Donato K, Villabona-Arenas CJ, Usme-Ciro JA, et al. Genomic epidemiology of severe acute respiratory syndrome coronavirus 2, Colombia. *Emerg Infect Dis.* 2020;26(12):2854-2862.

28. Lewandowski K, Xu Y, Pullan ST, et al. Metagenomic nanopore sequencing of influenza virus direct from clinical respiratory samples. *J Clin Microbiol.* 2020;58(1).

29. Lu J, Peng J, Xiong Q, et al. Clinical, immunological and virological characterization of COVID-19 patients that test re-positive for SARS-CoV-2 by RT-PCR. *EBioMedicine.* 2020;59.

30. Madi N, Al-Nakib W, Mustafa AS, Habibi N. Metagenomic analysis of viral diversity in respiratory samples from patients with respiratory tract infections in Kuwait. *Journal of Medical Virology.* 2018;90(3):412-420.

31. Maurier F, Beury D, Flechon L, et al. A complete protocol for whole-genome sequencing of virus from clinical samples: Application to coronavirus OC43. *Virology.* 2019;531:141-148.

32. Moore SC, Penrice-Randal R, Alruwaili M, et al. Amplicon-Based Detection and Sequencing of SARS-CoV-2 in Nasopharyngeal Swabs from Patients With COVID-19 and Identification of Deletions in the Viral Genome That Encode Proteins Involved in Interferon Antagonism. *Viruses-Basel.* 2020;12(10).

33. Nieto A, Pozo F, Vidal-García M, Omeñaca M, Casas I, Falcón A. Corrigendum: Identification of rare PB2-D701N mutation from a patient with severe influenza: Contribution of the PB2-D701N mutation to the pathogenicity of human influenza [Front. Microbiol. 8, 575 (2017)] doi: 10.3389/fmicb.2017.00575. *Front Microbiol.* 2017;8(JUN).

34. O'Flaherty BM, Li Y, Tao Y, et al. Comprehensive viral enrichment enables sensitive respiratory virus genomic identification and analysis by next generation sequencing. *Genome Res.* 2018;28(6):869-877.

35. Parker J, Chen J. Application of next generation sequencing for the detection of human viral pathogens in clinical specimens. *J Clin Virol.* 2017;86:20-26.

36. Perez-Sautu U, Wiley MR, Iglesias-Caballero M, et al. Target-independent high-throughput sequencing methods provide evidence that already known human viral pathogens play a main role in respiratory infections with unexplained etiology. *Emerging microbes & infections.* 2019;8(1):1054-1065.

37. Piralla A, Rovida F, Girello A, et al. Frequency of respiratory virus infections and next-generation analysis of influenza A/ H1N1pdm09 dynamics in the lower respiratory tract of patients admitted to the ICU. *PLoS ONE.* 2017;12(6).

38. Ren X, Hu Y, Yang F, et al. Clinical utility comparison of two benchtop deep sequencing instruments for rapid diagnosis of newly emergent influenza infections. *Clin Microbiol Infect.* 2015;21(3):290.e291-290.e294.

39. Roy S, Hartley J, Dunn H, Williams R, Williams CA, Breuer J. Whole-genome Sequencing Provides Data for Stratifying Infection Prevention and Control Management of Nosocomial Influenza A. *Clinical Infectious Diseases.* 2019;69(10):1649-1656.

40. Sarkale P, Patil S, Yadav PD, et al. First isolation of SARS-CoV-2 from clinical samples in India. *Indian J Med Res.* 2020;151(2):244-250.

41. Shah SJ, Barish PN, Prasad PA, et al. Clinical features, diagnostics, and outcomes of patients presenting with acute respiratory illness: A retrospective cohort study of patients with and without COVID-19. *EClinicalMedicine.* 2020.

42. Thi Kha Tu N, Thi Thu Hong N, Thi Han Ny N, et al. The Virome of Acute Respiratory Diseases in Individuals at Risk of Zoonotic Infections. *Viruses.* 2020.

43. Thorburn F, Bennett S, Modha S, Murdoch D, Gunson R, Murcia PR. The use of next generation sequencing in the diagnosis and typing of respiratory infections. *J Clin Virol.* 2015;69:96-100.

44. Tushir S, Kamanna S, Nath SS, et al. Proteo-Genomic Analysis of SARS-CoV-2: A Clinical Landscape of Single-Nucleotide Polymorphisms, COVID-19 Proteome, and Host Responses. *J Proteome Res.* 2021.

45. Wu X, Cai Y, Huang X, et al. Co-infection with SARS-CoV-2 and influenza a virus in patient with pneumonia, China. *Emerg Infect Dis.* 2020;26(6):1324-1326.

46. Xiao F, Sun J, Xu Y, et al. Infectious SARS-CoV-2 in feces of patient with severe COVID-19. *Emerg Infect Dis.* 2020;26(8):1920-1922.

47. Yadav PD, Potdar VA, Choudhary ML, et al. Full-genome sequences of the first two SARS-CoV-2 viruses from India. *Indian J Med Res.* 2020.

48. Yang J, Yang F, Ren L, et al. Unbiased parallel detection of viral pathogens in clinical samples by use of a metagenomic approach. *J Clin Microbiol.* 2011;49(10):3463-3469.

49. Zhang D, Lou X, Yan H, et al. Metagenomic analysis of viral nucleic acid extraction methods in respiratory clinical samples. *BMC Genomics.* 2018;19.

50. Zoll J, Rahamat-Langendoen J, Ahout I, et al. Direct multiplexed whole genome sequencing of respiratory tract samples reveals full viral genomic information. *J Clin Virol.* 2015;66:6-11.

51. Zou XH, Chen WB, Zhao X, et al. Evaluation of A Single-reaction Method for Whole Genome Sequencing of Influenza A Virus using Next Generation Sequencing. *Biomed Environ Sci.* 2016;29(1):41-46.

52. Zuckerman NS, Pando R, Bucris E, et al. Comprehensive analyses of SARS-CoV-2 transmission in a public health virology laboratory. *Viruses.* 2020;12(8).
